# Comparison of Vaginal Misoprostol with Foley Catheter for Cervical Ripening and Induction of Labor

**Published:** 2011

**Authors:** Fatemeh Vahid Roudsari, Sedigheh Ayati, Marzieh Ghasemi, Maliheh Hasanzadeh Mofrad, Mohamad Taghi Shakeri, Farnoush Farshidi, Masoud Shahabian

**Affiliations:** a *Women’s Health Research Center, Department of Obstetrics and Gynecology, Mashhad University of Medical Sciences, Ghaem Hospital, Mashhad, Iran.*; b *Department of Medicosocial, Biostatics Unit, Mashhad University of Medical Sciences, Ghaem Hospital, Mashhad, Iran.*; c *General Practitioner, Mashhad University of Medical Sciences, Cardio-Vascular Research Center, Mashhad, Iran.*

**Keywords:** Misoprostol, Foley catheter, Cervical ripening, Induction of labor

## Abstract

At times, despite an unripe cervix, induction of labor may be needed. In these cases, a safe and suitable method should be considered for cervical ripening and pregnancy termination. The aim of this study is the comparison of vaginal misoprostol with Foley catheter for cervical ripening and induction of labor. This randomized clinical trial was performed on 108 pregnant women who had referred to the teaching hospitals of Mashhad University of Medical Sciences during a time period of September 2007 to March 2008. These women were randomly divided into two groups: Misoprostol (including 49 patients) and Foley catheter (including 59 patients). For the first group, 25 microgram vaginal misoprostol was administered every 4 h up to maximum 6 doses. For the second group, Foley catheter 18 F, inflated with 50 cc of sterile water, was placed through the internal os of the cervix. Data was analyzed using SPSS software. p < 0.05 was considered statistically significant. Two groups were similar in the view of demographic characteristics, cesarean indications, maternal and fetal outcomes and neonatal outcomes. Vaginal delivery was significantly higher in misoprostol group (89.9 vs. 62.7, p < 0.01). The mean of delivery time was significantly shorter in misoprostol group (11.08 ± 5.6 vs. 13.6 ± 16.0 h, p < 0.05). In the cases of pregnancy termination and unripe cervix, two methods of misoprostol and Foley catheter were considered suitable, but it seemed that misoprostol decreases the delivery time and was needed for the cesarean section.

## Introduction

In the recent decade, there has been a considerable increase in the rate of labor induction, a common method in the termination of pregnancy. Approximately, 20% of all deliveries are initiated with this method (1). Labor induction is usually performed when the risks of continuing a pregnancy are more than the benefits of delivery. Undoubtedly, cervical ripening has a close relationship with the success rate of delivery. Different methods are used for labor induction. Induction of labor with oxytocin in the presence of a low Bishop score can not lead to vaginal delivery in a suitable period of time and also is followed by increasing rate of cesarean section. Hence, methods of cervical ripening that ripen the cervix in a short period of time play an important role in modern obstetrics (2). Although there are many proper methods for cervical ripening, there exists no agreement on the choice of best and most proper labor induction of cases with unripe cervix. Among these methods, cervical foley catheter and vaginal misoprostol (prostaglandin E1) are used for labor induction and cervical ripening (3-5). Since misoprostol is relatively cheap, stable at room temperature and has good effect, it is frequently used in obstetrics and gynecology for termination of pregnancy especially at third trimester (6).

In the study that was performed by Kashanian *et al*. Foley catheter with different balloon volumes were compared to oxytocin for cervical ripening and labor induction. They concluded that Foley catheter is a safe and suitable method for patients with an unfavorable cervix, and might reduce the duration of labor and increase the number of deliveries within 24 h; moreover, the larger balloon volume might improve these effects (7). Some other studies reported the same results (8, 9).

Adeniji *et al*. performed a study in 2006 to compare vaginal misoprostol and Foley catheter for cervical ripening. They reported that vaginal misoprostol was more effective to improve the scores of cervical length and consistency, while Foley catheter was better to improve the cervical os dilatation score during the preinduction cervical ripening (10). 

Fekrat *et al*. studied three methods of cervical ripening and labor induction with vaginal misoprostol and Foley catheter and a combination of these two methods. The duration between induction of labor and delivery was significantly lower in misoprostol group. They resulted that the combination of these two methods didn’t have more efficacy on cervical ripening (11). 

The aim of this study is the comparison of vaginal misoprostol with Foley catheter for cervical ripening and induction of labor.

## Experimental

This randomized clinical trial was performed on 110 pregnant women admitted to the labor ward for induction of labor. The study was conducted in the Department of Obstetrics, teaching hospitals, Mashhad University of Medical Sciences during a time period of September 2007 to March 2008.

The included criteria were gestational age > 37 weeks on the basis of last menstrual period (LMP) or sonography at first trimester, need to pregnancy termination for fetal or maternal indication, unfavorable cervix (Bishop score < 7), gestational diabetes mellitus, singleton pregnancy, reassuring fetal heart rate tracing, cephalic presentation, intact membranes, low-located placenta, and mild preeclampsia. Women were excluded from the study if any of the following criteria were encountered: hypersensitivity to prostaglandin, temperature > 38^˚^C, previous cesarean delivery or other uterine surgery, placenta previa, chorioamnionitis, vaginal bleeding, fetal distress, need to immediate delivery, macrosomy, and polyhydroamnios. They were randomly divided into two groups: 50 cases in misoprostol group (group 1) and 60 cases in Foley catheter group (group 2). At first, the method of the study was completely explained for them; if the written consent was obtained, they were entered the study. This study was approved by the ethics committee of mashhad university of medical sciences. 

For the first group, misoprostol tablet (Cytotec; Pfizer: SA Madrid) was used. 25 μg misoprostol was placed in the posterior fornix of the vagina; if needed, it was repeated up to 6 doses every 4 h. Vaginal examination was performed every 4 h; if the uterine contractions didn’t begin, the patient received another dose. In the presence of spontaneous and frequent contractions (about 40 -50 sec every 3 min), the next dose was not administered. If the effective uterine contractions didn’t begin 4 h after the last dose, oxytocin infusion was used.

For the second group, 18 F Foley catheters were placed through the cervix in the sterile condition. The balloon was inflated with 50 cc of sterile saline solution and pulled against the internal os of the cervix. In the absence of uterine contractions after 12 h, labor induction was done with oxytocin. At first, oxytocin infusion was used with a dose of 2 mLU/min, as required 2 mLU/min was increased every 20 min in order to effective uterine contraction (at least 3 contractions of 40 - 50 sec every 3 min). The maximum administered dose was 40 mLU/min. If there was inadequate uterine contraction or no progress in the active phase and fetal or maternal indication, cesarean delivery was performed for the patient. From each group, one patient was excluded from further analysis (due to the bad participation) and totally 49 cases in first group and 59 cases in second group completed the study.

All data were gathered prospectively with the use of questionnaire. Maternal demographic characteristics (maternal age, gestational age, parity, mode of delivery, first bishop score, neonatal apgar score) were recorded for both groups and then were compared. The indication for the induction and important outcomes of labor were recorded for each patient: the placement time of the Foley catheter or misoprostol, the expulsion time of Foley catheter, the amniotomy or spontaneous rupture time of membranes, the initiation time of oxytocin, the time of second stage of labor, and the delivery time. In this study, the main variable was the interval time from the first intervention to the time of delivery. During intervention, the patient was assessed for possible outcomes, uterine tachysystole (defined as ≥ 6 contractions every 10 min), and uterine hyperstimulation (continuing contractions more than 2 min). Fetal heart rate tracing was recorded every 15 min. 

Statistical analysis was performed using SPSS software (Version 11.5), and then comparison was made with X² test and Exact Fisher test for the qualitative data. After controlling the normality, Mann-Whitney U test and kruskal-wallis test was used if the normality did not fit and then independent t-test and ANOVA test was used if normality fitted to data. p < 0.05 was considered statistically significant. 

## Results and Discussion

In this study, a total of 108 pregnant women with indication for pregnancy termination were evaluated. They were randomizedly divided into two groups: 49 cases in misoprostol group as first group and 59 cases in Foley catheter group as second group. The studied groups were similar in the view of demographic characteristics including age, gestational age, parity, and Bishop score.

The mean and the standard variation of age in misoprostol group and Foley catheter was 24.3 ± 4.0 and 24.2 ± 5.0 (p > 0.1), respectively. Gestational age, in first group was 39.8 ± 1.4 weeks and in second group was 40 ± 0.9 weeks (p > 0.1). Parity, in first group was 1.3 ± 0.63, and in second group was 1.7±1.1 (p > 0.1). The Bishop score in misoprostol group was 2.7 ± 1.3 and in Foley catheter group was 2.0 ± 1.6 (p > 0.1).

As it was shown in Table 1, the rate of vaginal delivery in first group was 89.8% and in second group was 62.7%. The rate of vaginal delivery was significantly higher in misoprostol group (p < 0.01).

**Table 1:** The mode of delivery in the studied groups

**Groups **	**Misoprostol**	**Foley catheter**	**Total**
**Mode of delivery**	N (%)	N (%)	N (%)
**Vaginal delivery**	44 (89.8)	37 (62.7)	81 (75.0)
**Cesarean delivery**	5 (10.2)	22 (37.3)	27 (25.0)
**Total**	49 (100.0)	59 (100.0)	108 (100.0)

Table 2 Shows indications of cesarean delivery in the studied groups.

**Table 2:** Cesarean indications in the studied groups

**Groups**	**Misoprostol**	**Foley catheter**	**Total**
**Cesarean indications**	N (%)	N (%)	N (%)
**Fetal distress**	2 (4.0)	4 (6.8)	6 (5.5)
**No progress at first stage**	1 (2.0)	5 (8.5)	6 (5.5)
**No progress at second stage**	0 (0.0)	5 (8.5)	5 (4.6)
**Meconial amniotic fluid**	2 (4.0)	7 (11.9)	9 (8.3)
**Cord prolapse**	0 (0.0)	1 (1.7)	1 (0.0)

Regarding the results of the studied groups shown in Table 3, placental residue occurred in 2% of the first group patients, but no one of the second group were complicated by this outcome (p > 0.1). Tachysystole was observed in 2% of the misoprostol group patients and no one of the Foley catheter group (p > 0.1). 5% of the first group and 6% of the second group were complicated by atony after delivery (p > 0.1). The uterine hypertonisity defined as contractions lasted more than two min was observed in 2% of the first group and no one of 

As it was shown in Table 4, the mean of latent phase was 8.5 ± 5.1 h in misoprostol group and 10 ± 6.8 h in Foley catheter group and there was no significant difference between the two groups (p > 0.1).The mean of time to delivery was 11.08 ± 5.6 h in first group, and 13.6 ± 16.9 h in second group that was significantly shorter in group 1 (p < 0.05).

**Table 3:** Pregnancy outcomes in the studied groups

**Groups**	**Misoprostol**	**Foley catheter**	**p-value**
**Outcomes**	N (%)	N (%)	N (%)
**Residue**	1 (2.0)	0 (0.0)	> 0.1
**Tachysystol**	1 (2.0)	0 (0.0)	> 0.1
**Atony**	3 (6.0)	3 (5.0)	> 0.1
**Uterine hypertonisity**	1 (2.0)	0 (0.0)	> 0.1

**Table 4:** The mean of parturiation phase in the studied groups

Groups	Mean ± standard deviation (h)		p-value
	Misoprostol	Foley catheter	
Latent phase	8.5 ± 5.1	10 ± 6.8	> 0.1
Time to delivery	11.08 ± 5.6	13.6 ± 16.9	< 0.05

Table 5 shows the neonatal outcomes. In first group, the mean of neonatal birthweight was 3182 ± 430 g, first min Apgar was 8, five min Apgar was 9, and the rate of meconial amniotic fluid expulsion was 10%. In second group, the mean of neonatal birthweight was 3323.8 ± 353 g, first min Apgar was 8, five min Apgar was 9, and the rate of meconial amniotic fluid expulsion was 5%.

**Table 5:** Neonatal outcomes in the studied groups

**Groups**	**Misopro stol**	**Mean ± standard deviation**	**p-value**
**Birth weight (g)**	3182 ± 430	3323.8 ± 353	< 0.05
**First min apgar**	8	8	1
**Five min apgar**	9	9	1
**Meconial amniotic fluid**	5 (10%)	3 (5%)	> 0.1

In this study, two methods of cervical ripening and labor induction with vaginal misoprostol and Foley catheter were compared. The results of the present study show that the rate of success in misoprostol group was more than Foley catheter group. In a study, Fekrat *et al*. evaluated three methods of cervical ripening and labor induction with vaginal misoprostol and Foley catheter and the combination of theses two methods. They reported that vaginal misoprostol was more effective than two other methods (11). A systematic review study performed by Hofmeyr *et al*. evaluated intra-vaginal misoprostol and other conventional intra-vaginal prostaglandins and showed that the misoprostol is more effective for cervical ripening and labor induction (12). Some other investigators obtained the same results (13-16). However, in a few studies no differences were found between Foley catheter and misoprostol for cervical ripening and induction of labor (17). Their findings are against the results of the present study. The most important cause for this result may be lower repeated doses of misoprostol that was used in their study compared with this study (which was 25 μg every 4 h misoprostol up to 6 doses). The American College of Obstetricians and Gynecologists recommends a maximum dose of 50 μg every 6 h for cervical ripening and induction of labor (18).

In this research, the mean time to delivery was significantly shorter in misoprostol group rather than the Foley catheter group. Adeniji *et al*. performed a study on 50 cases in misoprostol group and 46 cases in Foley catheter group. They reported that the duration of cervical ripening was shorter in misoprostol group which is in consistent with the result of the present study (19). The number of the studied patients in their study was similar to ours. In the other study performed by Adeniji *et al*. on 102 patients in misoprostol group and 96 cases in Foley catheter group, it was indicated that the misoprostol was more effective in improving the scores of cervical length and consistency, while Foley catheter was better in improving the cervical os dilatation score during preinduction cervical ripening (10). Hill *et al*. in 2008 reported that the duration between induction and delivery in Foley catheter plus oral misoprostol group was significantly shorter than that of vaginal misoprostol group (6). Dalui *et al*. compared the Foley catheter and prostaglandin gel E2 for cervical ripening and the results of misoprostol group were more successful (20). Type of prostaglandin and method of administration may be the cause of difference between the two studies.

In the present study, the rate of vaginal delivery was significantly higher in misoprostol group, but in the study of Afolabi *et al*. which was performed in 2005 on 100 pregnant women, it was shown that there was no significant different between the rate of vaginal delivery in studied groups (21). In their study, amniotomy and then labor induction were performed after cervical ripening with increased dose of oxytocin which may be premature amniotomy at this stage and be the cause of these differences. Their findings were in harmony with the results of Kramer *et al*. (22). Barrileax *et al*. that compared oral misoprostol with Foley catheter didn’t report significant difference between the two groups in the view of the rate of vaginal delivery (2).

In the present research, there was no significant difference between the two groups in the view of cesarean inductions. Moreover, both groups were similar in the view of the first and fifth min neonatal Apgar score. Some other studies reported the same results (6, 11).

## Conclusion

The results of the present study indicate that vaginal misoprostol improves the process of delivery and increases the rate of vaginal delivery in the cases of unripe cervix. However, more studies with higher volume samples can be led to justify these results.
